# The Dynamics of Antibody Titres Against SARS-CoV-2 in Vaccinated Healthcare Workers: A Systemic Literature Review

**DOI:** 10.3390/vaccines12121419

**Published:** 2024-12-16

**Authors:** Vilija Gurkšnienė, Tadas Alčauskas, Fausta Majauskaitė, Ligita Jančorienė

**Affiliations:** Faculty of Medicine, Vilnius University, 03101 Vilnius, Lithuania; vilija.gurksniene@santa.lt (V.G.); fausta.majauskaite@santa.lt (F.M.); ligita.jancoriene@santa.lt (L.J.)

**Keywords:** antibodies, COVID-19, healthcare workers, vaccination

## Abstract

**Background and Objectives:** Given that COVID-19 vaccination is a relatively recent development, particularly when compared to immunisation against other diseases, it is crucial to assess its efficacy in vaccinated populations. This literature review analysed studies that monitored antibody titres against SARS-CoV-2 in healthcare workers who received COVID-19 vaccines. **Methods:** Using the PICO (Population, Intervention, Comparators, Outcomes) model recommended in the PRISMA (Preferred Reporting Items for Systematic Reviews and Meta-Analyses) guidelines we included 43 publications which analyse antibody dynamics following primary vaccination, the effects of booster doses, and the influence of factors such as COVID-19C infection, age, and sex on antibody kinetics. **Results:** All the studies demonstrated a strong immunogenic response to the vaccines. Re-gardless of the vaccine used, over 95% of the pre-vaccination seronegative population be-came seropositive in all studies. Depending on the sampling intervals provided by the re-searchers, antibody levels were quantitatively highest during the first three months after vaccination, but levels inevitably declined over time. The monthly decline in antibodies observed in all these studies highlighted the necessity for booster doses. Studies analysing the impact of revaccination on antibody dynamics have confirmed that revaccination is an effective tool to boost humoral immunity against SARS-CoV-2. An-tibodies appear to persist for a longer period of time after revaccination, although they are subject to similar factors influencing antibody dynamics, such as age, comorbidities, and exposure to COVID-19. In addition, heterogeneous revaccination strategies have been shown to be more effective than homogeneous revaccination. **Conclusions:** Our review demonstrated that antibody levels against SARS-CoV-2 inevitably decline after vaccination, leaving the question of ongoing booster strategies open. The studies reviewed provided evidence of the effectiveness of booster vaccination, despite differences in age, sex, and prior COVID-19 infection. This suggests that repeated vaccination remains a highly effective method for mitigating the continued threat posed by COVID-19.

## 1. Introduction

In December 2019, a novel virus, SARS-CoV-2, responsible for COVID-19, was identified in China’s Hubei province. The disease rapidly spread across the globe. According to the World Health Organization (WHO), as of 15 September 2024, there were nearly 776.3 million cases of COVID-19 worldwide, with an estimated death toll exceeding 7 million [1]. Healthcare workers are among the most at risk of contracting SARS-CoV-2 [2,3]. In the United Kingdom, serological data from 2063 hospital staff indicated that healthcare workers face a threefold higher risk of COVID-19 infection compared to the general population [4]. Studies highlight that emergency medical personnel are one of the most vulnerable groups among healthcare workers, accounting for 31–33% of all infected healthcare staff [5,6]. The risk of infection is also notably high among general nurses, with various reports suggesting that this group represents between 48 and 55% of hospitalised healthcare workers [7,8].

In September 2020, the WHO Strategic Advisory Group of Experts on Immunisation (SAGE) issued guidelines on vaccination against COVID-19, recommending that healthcare professionals be prioritised for immunisation [9]. Following the introduction of COVID-19 vaccination for healthcare workers, the first data on vaccine efficacy in this group began to emerge. In the United Kingdom, a study involving 23,324 healthcare workers found that the incidence of COVID-19 in unvaccinated individuals was 14 cases per 10,000 person-days, whereas among those who had received a second dose of the BNT162b2 mRNA vaccine, the incidence dropped to just 4 cases per 10,000 person-days [10]. Similar studies conducted in Tel Aviv, Israel, and Boston, United States, also reported significantly lower rates of COVID-19 infection post-vaccination [11,12].

However, even with positive immunisation outcomes among healthcare workers, a small risk of contracting COVID-19 or becoming a carrier of SARS-CoV-2 remains. At Sheba Medical Centre, Israel’s largest healthcare facility, 39 cases were identified among 1497 fully vaccinated workers who were tested using PCR. Of these, 33% were asymptomatic carriers and 67% experienced mild symptoms. All infected workers had lower antibody titres against SARS-CoV-2 in serological tests compared to uninfected groups [13].

Since COVID-19 vaccination is a relatively recent development, especially when compared to vaccines for other diseases, and new subtypes of the virus are continuing to emerge, it is essential to monitor and evaluate the efficacy of vaccination in immunised individuals. Additionally, COVID-19 vaccines are of particular interest because of the new technology used. Both Pfizer and Moderna vaccines contain a nucleoside-modified mRNA that encodes the SARS-CoV-2 spike glycoprotein and is delivered in lipid nanoparticles for more efficient delivery into host cells. The mRNA specifically encodes the S2-P antigen, consisting of the SARS-CoV-2 glycoprotein with a transmembrane anchor. The vaccine’s goal is to elicit both B- and T-cell responses against the spike protein. The potent lipid-nanoparticle delivery system used by the vaccine in combination with the use of modified nucleotides that avoid early activation of interferon-associated genes are unique features that contribute to its efficacy. The mRNA vaccine is intended to produce prolonged protein expression, induction of antigen-specific T-follicular helper cells, and activation of germinal centre B-cells [14].

During the pandemic, SARS-CoV-2 evolved through mutations in its genetic material, leading to the emergence of variants with altered transmissibility, immune evasion capabilities, and vaccine effectiveness. A recent analysis demonstrated that even with high vaccination rates among healthcare personnel, SARS-CoV-2 reinfections were frequent during the Omicron transmission period [15]. In post-Omicron studies, working in healthcare facilities was identified as the most influential variable in all risk factor analyses, emphasising the importance of comprehending infection patterns within specific hospitals and departments [16]. This is particularly important for healthcare workers caring for immunosuppressed patients or those on the frontlines of the COVID-19 response. The aim of this systemic literature review was to assess the dynamics of antibody titres in healthcare workers vaccinated against COVID-19.

## 2. Materials and Methods

Using the PICO (Population, Intervention, Comparators, Outcomes) model recommended in the PRISMA (Preferred Reporting Items for Systematic Reviews and Meta-Analyses) guidelines [17] (Table 1), we formulated the primary question of this systematic review: What are the antibody titres against SARS-CoV-2 in healthcare workers vaccinated with the COVID-19 vaccine at different time points post-vaccination?

Articles meeting all the following criteria were included in the systemic review:

1. The study population consisted of healthcare workers.
2. The study population had been vaccinated with one of the following COVID-19 vaccines: Pfizer-BioNTech, Oxford–AstraZeneca, Moderna, or Janssen.
3. The study population was periodically tested for antibodies against SARS-CoV-2 following vaccination.
4. Antibody levels were measured at intervals over a minimum duration of 3 months.
5. The study was published in English.
6. The study was quantitative in nature.
7. The research was conducted in Europe.
8. The research publication was accessible via the Vilnius University intranet.

The following exclusion criteria were applied:

1. The study population was tested for cellular immunity.
2. The study population was assessed for the effects of combining COVID-19 vaccines with other vaccines.
3. The study was a clinical trial, literature review, or pre-print.

European studies were chosen because Europe had a unified COVID-19 vaccination strategy, choosing to use vaccines from Pfizer-BioNTech, Oxford-AstraZeneca, Moderna, or Janssen. This purification of the studies allowed for a more detailed look at the behaviour of antibody response in the context of a similar immunoprophylactic strategy.

The search for publications suitable for inclusion in the review was conducted using the computerised bibliographic MEDLINE database (via PubMed). The search took place between 1 June 2024 and 31 August 2024. The keywords and keyword combinations used were as follows: (“vaccination” OR “immunoprophylaxis”) AND (“healthcare workers” OR “doctors” OR “healthcare specialists” OR “hospital workers”) AND (“antibodies” OR “immunoglobulins”). Advanced filters applied included English language and publication dates between 2020 and 2024.

**Figure 1:**
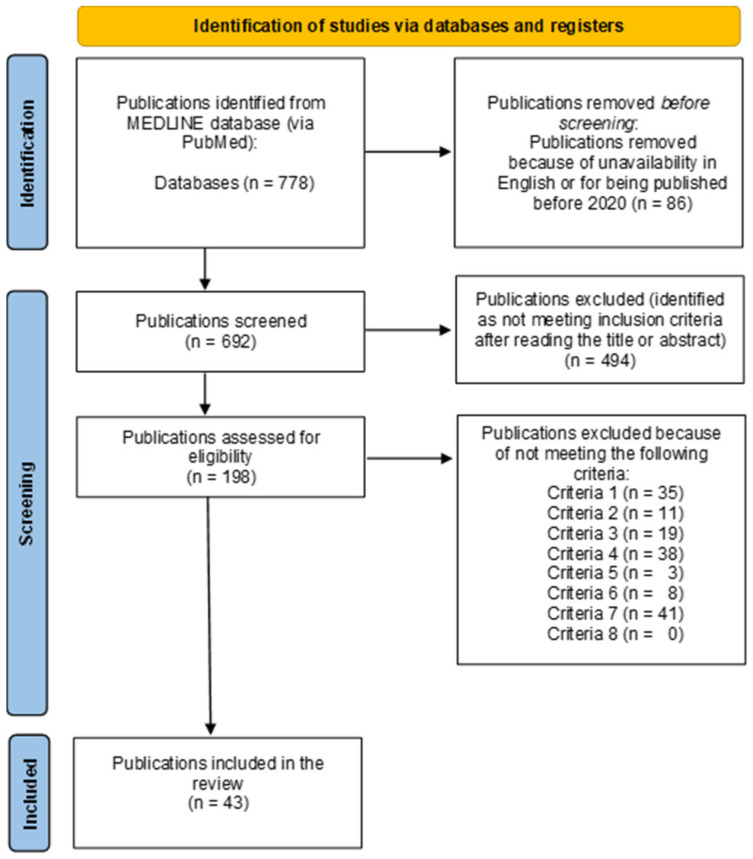
The publication selection process.

The selection of publications was based on predefined inclusion and exclusion criteria. The process for determining whether scientific articles met the criteria involved two stages. In the first stage, the titles and abstracts of 692 articles, retrieved through keyword searches, were reviewed. In this sage, two reviewers (V.G. ang T.A.) looked independently for eligible publications. From this, 198 articles were identified as potentially meeting the criteria. In the second stage, full-text articles were read in detail, and studies were assessed for compliance with the criteria. The selected studies were read by three reviewers independently (V. G., T. A. and F. M.). A total of 155 articles were rejected. The reasons for rejection were as follows: the research population was not healthcare workers (*n* = 35); the research population had not been vaccinated with the specified vaccines (*n* = 11); the research population had not been tested for antibodies against SARS-CoV-2 (*n* = 19); the duration of antibody monitoring was less than 3 months (*n* = 38); the study was not available in English (*n* = 3); the research was not quantitative in nature (*n* = 8); or the research had been conducted outside of Europe (*n* = 41). Ultimately, 43 studies were included in the review. After eligible studies were obtained, each of the three reviewers reviewed papers and divided them into two groups: those analysing antibody dynamics before booster doses and those analysing the effect of booster doses on antibody kinetics. Articles describing antibody kinetics at different time points by quantitatively measuring antibody concentrations were categorised according to the types of antibodies measured. Articles measuring the same antibody type were compared. Information was also collected on which factors (e.g., exposure to COVID-19 infection, age, or sex) had an impact on the antibody dynamics described in the articles. The risk of whether the results may have been influenced by vaccine manufacturers paying for the publication of an article, specifically when one vaccine was presented as being more effective than another, was assessed when the authors reviewed the sources of funding for the articles. The selection of articles is illustrated in Figure 1.

The data from the included publications are summarised in Table S1, which can be downloaded from the Supplementary Materials section. The following information was collected for each publication analysed: the main author of the study, the year of publication, the country where the study was conducted, the size of the research group, the duration of antibody monitoring, the fact of booster effect analysis, the fact of COVID-19 exposure effect on antibody kinetics analysis, the fact of age and sex effects on antibody kinetics analysis, and the fact of heterogenous vaccination scheme analysis.

## 3. Results

The review of studies that met the inclusion criteria considered antibody kinetics following vaccination with COVID-19 vaccines and the immunogenicity of the vaccines themselves. The results of the review are presented under the following sub-headings: “Antibody dynamics after primary vaccination” and “The effect of booster doses”.

### 3.1. Antibody Dynamics After Primary Vaccination

We identified 21 studies that analysed antibody dynamics following initial COVID-19 vaccination (without booster doses). Of these, five studies monitored the research population for three months (28–22), one monitored for four months (23), one monitored for five months (24), eight monitored for six months (25–32), one monitored for seven months (33), and five monitored for eight months (34–38).

#### 3.1.1. Period of 3 Months

Anastassopoulou et al. described the kinetics of the anti-SARS-CoV-2 IgG anti-RBD antibody responses following two doses of the Pfizer-BioNTech BNT162b2 vaccine [18]. In the first period (3–4 weeks after vaccination), high anti-RBD IgG titres with an average value of 58,697 index units were measured. Females had higher titres (average: 60,967) compared to males (average: 54,325), and the 21–30 age group exhibited the highest mean titre levels, averaging 76,987, with a decline observed with increasing age. In the second period (3 months after vaccination), titres declined substantially, averaging 21,987 units across all participants. The differences between sex and age groups remained the same. The decreasing anti-RBD trend was also seen by Oliveira-Silva et al. [19]. In this study, prior to vaccination, most participants showed non-reactive IgG levels; however, 15 days after vaccination, median IgG levels spiked to approximately 21,700 AU/mL. After 3 months, titres dropped significantly to a median of 3200 AU/mL, indicating a 6.3-fold reduction from the 15-day period. Females and younger participants had consistently higher IgG levels, both immediately after vaccination and at the 3-month mark.

Salvagno et al. described the kinetics of total anti-SARS-CoV-2 anti-S antibodies during the 3-month period [20]. Both seropositive and seronegative individuals showed a peak in antibody levels after the second vaccine dose, followed by a decline by three months post-first dose. The decline was more pronounced in the seropositive group. Among seronegative individuals, those aged >65 years showed significantly lower antibody levels at both 30 days and 3 months post-first dose compared to younger individuals. However, no significant differences in antibody levels between sexes were observed at either time point. The study by Van Elslande et al., which examined anti-S IgG antibodies after receipt of two doses of the BNT162b2 vaccine, noted that participants with prior SARS-CoV-2 infection had significantly higher anti-S IgG levels at all time points compared to those without prior infection [21]. Antibody levels declined significantly from 6 weeks to 3 months after the first dose in both groups but remained higher in the previously infected group. Three months after vaccination, previously infected vaccinated participants showed considerably higher median antibody titres than those with natural infection (24.5 times higher) and unvaccinated healthcare workers (4.6 times higher). Visci et al., who also investigated the humoral immune response to SARS-CoV-2 vaccination by measuring anti-S IgG antibodies, observed a decline in antibody levels over a period of 3 months, which was slower in female healthcare workers, younger individuals, and those who had prior COVID-19 infection [22].

Summary of the period: From the studies analysed, it is clear that the strongest immune response to vaccines is observed in the first weeks after vaccination, while the first drop in antibody titres can be detected as early as one month later. This decline continues until 90 days after vaccination.

#### 3.1.2. Period of 4–5 Months

A study by Brisotto et al. tracked anti-RBD levels after vaccination with either the Pfizer-BioNTech BNT162b2 or Moderna mRNA-1273 vaccine for 4 months [23]. As in previous studies, significant antibody decay was observed, with median levels dropping from 559.8 AU/mL (IQR: 359.7–845.7) at one month to 92.7 AU/mL (IQR: 65.1–148.6) at four months (*p* < 0.001). In this study, decline was independent of age and sex. Antibody levels were inversely correlated with age, and individuals with a history of prior SARS-CoV-2 infection had significantly higher antibody levels. Those who received the mRNA-1273 vaccine tended to have higher antibody levels than those who received BNT162b2 (statistically significantly higher levels in the case of those without prior infection).

Anti-S IgG levels 5 months after vaccination with the Pfizer-BioNTech BNT162b2 vaccine were examined by Cangemi et al. [24]. The median anti-S IgG level was 693 AU/mL (IQR: 394–800 AU/mL) after 1 month. Levels above 800 AU/mL were associated with prior COVID-19 infection and inversely associated with age, smoking, and autoimmune diseases. A substantial decline in anti-S IgG levels was observed after 5 months, with a median decrease of 72% (IQR: 60–82%). The median level at 5 months was 187 AU/mL (IQR: 81–262 AU/mL). This decrease was significantly associated with male sex, older age, smoking, and hypertension and inversely associated with prior COVID-19 infection.

Summary of the period: Although there is a clear downward trend in antibody titres when analysing antibody dynamics over a 4–5-month period, it is during this period that co-factors leading to longer or shorter antibody persistence begin to stratify. The authors identified factors influencing antibody dynamics, such as history of COVID-19 infection, age, smoking, and comorbidities.

#### 3.1.3. Period of 6 Months

Bayart et al. measured anti-SARS-CoV-2 total antibodies, anti-SARS-CoV-2 IgG antibodies, and anti-SARS-CoV-2 neutralising antibodies [25]. A significant decline in antibodies was observed six months post-vaccination in both seronegative (previously uninfected) and seropositive (previously infected) individuals. The decline was more pronounced for neutralising antibodies than for total antibodies or IgG antibodies. Approximately 45% of subjects tested negative for neutralising antibodies at day 180. The concentrations of neutralising antibodies in the seronegative group were 1955 (1287–2622) at day 28 and dropped to 26.1 (20.1–32.1) at day 180. Total antibody levels decreased by 55.4% in seronegative individuals and 74.8% in seropositive individuals at day 180. IgG antibody levels decreased by 89.6% in seronegative individuals and 79.4% in seropositive individuals at day 180. The half-life of IgG was 21 days in seronegative individuals and 53 days in seropositive individuals. Meanwhile, Collatuzo et al. analysed the determinants of anti-S IgG antibody levels six months after a two-dose COVID-19 vaccination in a large multicentre European cohort of healthcare workers [26]. Six months post-vaccination, a serological response was detected in 99.6% of participants. Several factors predicted higher IgG titres in this study: women had a higher antibody response than men (relative risk: 1.10, 95% confidence interval (CI): 1.00–1.21). Participants with a history of COVID-19 infection also showed significantly higher antibody levels (relative risk (RR): 2.26, 95% CI: 1.73–2.95). mRNA vaccines were associated with higher antibody levels compared to viral vector vaccines (*p* < 0.001). Counterintuitively, younger age and a shorter interval since the last vaccine dose were associated with lower antibody levels.

Anti-RBD IgG levels in a 6-month period were measured in a study by Ðakovi’c Rode et al. [27]. Participants without a history of COVID-19 had much lower anti-RBD IgG levels than those with a history of prior infection. The mean concentrations after three weeks were 873.5 AU/mL (402.9–1753.3) and 966.0 AU/mL (583.6–1431.8) after six months in the COVID-19-naïve group, while those who had been infected previously had levels of 14,280.2 AU/mL (6913.4–22,347.7) and 1465.2 AU/mL (1021.0–3559.1) in the corresponding periods. In a study by Krintus et al., which also measured anti-RBD IgG levels, an analogous trend was noticed: the 3 months post-vaccination median anti-RBD IgG concentration was 1145 BAU/mL (IQR: 543–2095), and the 6 months post-vaccination median anti-RBD IgG titre decreased significantly to 225 BAU/mL (IQR: 100–510) [28]. Factors associated with higher antibody levels were fever after both first and second doses, prior COVID-19 infection, and muscle pain after the first dose of the vaccine.

The study by Fernández-Rivas et al. measured anti-SARS-CoV-2 trimeric IgG antibodies during the same period after vaccination [29]. In the initial testing, 98.04% of healthcare workers showed positive results for serological testing (≥33.8 BAU/mL). Only 1.96% were negative. The median BAU/mL was higher in vaccinated patients with no infection (1260 BAU/mL; 465–2080) compared to infected patients (661 BAU/mL; 361–2080). Symptomatic individuals had lower antibody levels than asymptomatic individuals during the six-month follow-up period.

Infantino et al. measured anti-S1 antibodies [30]. The study followed 57 healthcare workers for six months after their second dose of the BNT162b2 vaccine. One month after the second dose, the median anti-S1 IgG level was 1452 BAU/mL (IQR: 980–1632); three months after the second dose, the median anti-S1 IgG level was 762 BAU/mL (IQR: 568–930); and six months after the second dose, the median anti-S1 IgG level was 104 BAU/mL (IQR: 64–184). The decrease over six months was 92.8%. However, the study did not find a significant correlation between age or gender and antibody levels. Oliveira-Silva et al. presented similar results for anti-S IgG antibodies [31]. Initially, most participants were seronegative (median: 6.8 AU/mL, IQR: 6.8–6.8). Fifteen days post-second dose, a robust IgG response was observed in 97.6% of participants: the median IgG level was 21,300 AU/mL (IQR: 13,300–33,000). Six months post-second dose, the median IgG concentration decreased to 1000 AU/mL (IQR: 640–1600). Only 3.3% of participants still had levels above 4160 AU/mL. Factors associated with higher and more persistent antibody levels were female gender, younger age group, and pre-existing SARS-CoV-2 antibodies. Ramos et al. reported that six months post-second Pfizer-BioNTech dose, 74% of participants had detectable neutralising antibodies against the Wuhan/UK variant and 47% of participants had detectable neutralising antibodies against the South African/Brazil variants [32]. Specific IgG levels (anti-S1, anti-RBD, and anti-S2) varied among participants, but higher levels correlated with a greater likelihood of having detectable neutralising antibodies. This indicates that high total antibody levels are important predictors of neutralising antibody production.

Summary of the period: A further decline in antibody titres was observed 6 months after vaccination, but a debate has started to develop on the underlying factors. Evidence has emerged that age and gender do not necessarily lead to shorter persistence of antibody titres. In addition, the impact of antibody titres on the incidence of COVID-19 has started to be analysed, with the discovery that symptomatic individuals have lower antibody levels than asymptomatic ones. In addition, differences in antibody dynamics began to emerge between individuals vaccinated with different vaccines, suggesting that mRNA-based vaccines may help to maintain humoral immunity against SARS-CoV-2 for longer.

#### 3.1.4. Period of 7–8 Months

Mueller T. described a study that measured anti-RBD antibodies for 7 months after the administration of the BNT162b2 vaccine [33]. The anti-RBD antibody concentration decreased from 2120 U/mL (789–2501 U/mL) after five weeks to 634 U/mL (107–1553 U/mL) after 7 months. Anti-RBD antibodies were measured for 8 months by Gil-Manso et al. and Golec et al. [34,35]. Both mRNA-1273 and BNT162b2 vaccines in Manso et al.’s study induced high levels of anti-RBD IgG antibodies. mRNA-1273 induced significantly higher levels (median: 3625.70 BAU/mL) compared to BNT162b2 (median: 2053.47 BAU/mL). At day 240 post-vaccination, antibody levels decreased significantly for both vaccines. However, mRNA-1273 still maintained higher levels (median: 312.14 BAU/mL) than BNT162b2 (median: 126.47 BAU/mL). The factors that impacted antibody kinetics were vaccine type—mRNA-1273 consistently induced higher and more durable IgG anti-RBD antibody responses than BNT162b2—and age—on day 30, younger individuals (20–29 years old) had higher antibody levels regardless of vaccine type. However, by day 240, this difference was less pronounced, but the mRNA-1273 group showed sustained higher levels in older age groups, contrasting with the BNT162b2 group, where antibody levels declined more significantly in older individuals, particularly men. No significant overall difference was observed between genders in antibody levels or waning rates. The presence of comorbidities also did not significantly affect antibody levels or waning rates. Golec et al., who observed similar results, reported several other factors which influenced the persistence of anti-RBD IgG antibodies eight months after the second vaccination dose: participants with a history of SARS-CoV-2 infection before vaccination were significantly more likely to maintain detectable IgG levels at eight months (a 7-fold increase in the chance of maintaining long-term seropositivity); women showed a significantly higher likelihood of maintaining long-term immunity (six times more likely than men); and higher predicted muscle mass was associated with a greater chance of maintaining long-term seropositivity, while a higher body fat mass (BFM) was associated with a reduced chance. In this study, smokers had a significantly lower chance of maintaining long-term seropositivity compared to non-smokers.

A study by Serrano et al. measured anti-S1 IgG and anti-S IgM antibodies [36]. At two months post-vaccination, all 477 participants had detectable anti-S1 IgG. The mean concentration was significantly higher in the Moderna group (18,192 AU/mL) than in the Pfizer group (10,441 AU/mL). At eight months, all participants remained positive, but the mean IgG levels decreased significantly (by approximately 11,025 AU/mL). The decrease was greater in Moderna-vaccinated individuals. At two months post-vaccination, only 110 (23.06%) participants had detectable anti-S IgM. The mean index was higher in the Moderna group. At eight months, data on IgM levels were not provided. The study identified several factors influencing antibody kinetics: Moderna consistently induced higher anti-S1 IgG levels compared to Pfizer at both two and eight months post-vaccination; participants with a history of previous SARS-CoV-2 infection had significantly higher anti-S1 IgG levels at two months compared to those without a history of infection. This difference was observed with both vaccines, though it was more pronounced with Moderna. Antibody levels decreased more rapidly in participants under 30 years old at the eight-month time point. The study noted that while both vaccines induced a persistent IgG response at both time points, a substantial decrease in antibody levels was observed over the eight-month period, suggesting the need for revaccination. Wolszczak-Biedrzycka et al. similarly reported that at eight months post-second dose, the median antibody level was significantly higher in the group with a history of prior SARS-CoV-2 infection compared to the group without prior infection [37]. Women in this study showed significantly higher antibody levels than men, and individuals under 50 years of age tended to have higher antibody levels than those over 50. Inchingolo et al. reported one additional factor connected with the kinetics of anti-S IgG antibody titres: individuals with blood type O (regardless of Rh factor) consistently displayed the highest average antibody levels across all time points over eight months [38].

Summary of the period: The 7–8-month period finalises the assessment of the antibody response elicited by the initial vaccination schedule, clarifying previously identified factors influencing antibody dynamics, such as age, comorbidities, and the fact of having been exposed to COVID-19. The further decline in antibody titres opened the question of revaccination strategies.

#### 3.1.5. Summary of Antibody Dynamics After Primary Vaccination

All the studies demonstrated a strong immunogenic response to the vaccines. Regardless of the vaccine used, over 95% of the pre-vaccination seronegative population became seropositive in all studies. Depending on the sampling intervals provided by the researchers, antibody levels were quantitatively highest during the first three months after vaccination, but levels inevitably declined over time. The monthly decline in antibodies observed in all these studies highlighted the necessity for booster doses.

### 3.2. The Effect of Booster Doses

In the literature analysis, we identified 22 studies that investigated antibody dynamics following the administration of booster doses [39,40,41,42,43,44,45,46,47,48,49,50,51,52,53,54,55,56,57,58,59,60]. All these studies observed a clear enhancement of humoral immunity when subjects received the third and/or fourth booster dose.

Following the administration of booster doses, a notable increase in antibody levels, specifically IgG, has been documented across several studies. For instance, after the BNT162b2 vaccine booster, participants exhibited a significant increase in anti-SARS-CoV-2 IgG levels, reaching a peak at approximately 30 days post-booster, with levels then declining over the subsequent months [51]. The kinetics observed typically show an initial sharp rise in antibody titres, followed by a gradual decline. This pattern is consistent with findings from other studies which indicate that antibody levels can drop significantly within months following booster doses [59].

Augustinussen et al. highlighted that booster vaccination not only induces a noticeable increase in antibody levels but also equalises the humoral response, regardless of differences in the initial vaccination regimens [54]. Guibert et al. presented similar findings, suggesting that a booster dose standardises the level of effectiveness and immunogenicity across both heterologous and homologous COVID-19 vaccine regimens [44].

Additionally, boosters significantly extend the period during which high antibody levels are detectable. This finding is supported by De Pace et al., who demonstrated that high antibody titres after the initial vaccination can be detected up to 12 months, and a clinically significant protective humoral response against COVID-19 can be observed up to 20 months after the last booster dose [43]. Lorent et al. suggested a similar protective effect, showing that no new SARS-CoV-2 infections occurred in healthcare workers up to 10 months after booster vaccination [40].

Studies comparing heterologous and homologous booster strategies unanimously demonstrated the superiority of heterologous vaccination schemes. Serra N et al. showed that mixed mRNA combinations induce higher antibody levels [56]. Gerhards et al. also indicated the advantage of a mixed mRNA-/vector-based combination compared to a pure vector-based vaccination regimen [50].

The factors impacting the efficacy of boosters are similar to those which affect the antibody kinetics after initial vaccination. Individuals with a history of SARS-CoV-2 infection showed a more robust antibody response to booster doses compared to those who were naïve [39,40,41,59]. This suggests that previous exposure to the virus can enhance the vaccine-induced immune response, leading to higher and more sustained antibody levels post-booster [52]. Different age groups respond variably to booster vaccinations. Young individuals generally demonstrate higher antibody titres compared to older populations; however, older adults may experience a slower decline in antibody levels post-booster [51]. Gender differences have also been observed, with females showing higher initial antibody responses than males, although the decline rates did not differ significantly [59].

The study by Zurac et al. reported a 2.7-fold increase in IgG levels immediately after the booster, with a significant decline over three months, highlighting the importance of timely administration of subsequent doses to maintain immunity [59]. In contrast, the analysis by Grassi et al. suggested that, although there was a decline, a significant proportion of participants maintained a protective level of IgG antibodies up to 150 days post-booster [51].

The research conducted on the persistence of neutralising antibodies found that breakthrough infections could still occur even in vaccinated individuals, stressing the necessity for ongoing surveillance and potentially additional booster doses, especially in light of emerging variants like Omicron [42].

Lastly, the investigation into the impact of comorbidities demonstrated that individuals with underlying health conditions may exhibit different antibody kinetics, reinforcing the need for tailored vaccination strategies for these populations [45].

Summary of the Analysis of the Effect of Booster Doses

Studies analysing the impact of revaccination on antibody dynamics have confirmed that revaccination is an effective tool to boost humoral immunity against SARS-CoV-2. Antibodies appear to persist for a longer period of time after revaccination, although they are subject to similar factors influencing antibody dynamics, such as age, comorbidities, and exposure to COVID-19. In addition, heterogeneous revaccination strategies have been shown to be more effective than homogeneous revaccination.

## 4. Discussion

Our review indicates that antibody levels against SARS-CoV-2 wane over time. In some cases, the decline in humoral markers is more rapid, such as when an individual is vaccinated while being COVID-19-naive. We argue that this phenomenon can be explained based on general immunological principles.

Some studies have shown that human immunoglobulin G (IgG) has a half-life of 17.5–26 days [61,62,63]. A few months following infection or vaccination, the rate of antibody decline slows, as free immunoglobulins are cleared from circulation through IgG catabolism [64]. Amanna and Slifka suggest that during the early phase after infection or vaccination, the humoral response relies on two mechanisms: one that is memory B-cell-dependent and another that is memory B-cell-independent. As antigen levels in the bloodstream decrease over time, the memory B-cell-dependent mechanism wanes, leaving only long-lived plasma cells to continue synthesising antigen-specific immunoglobulins. This explains the periodic decline in antibody levels, as long-lived plasma cells produce antibodies at a much slower rate than short-lived plasma cells [65]. Additionally, Farber et al. propose that there is a homeostatic balance between maintaining effective humoral immune readiness and conserving metabolic resources by avoiding excessive production of immunoglobulins when the pathogen is absent [66].

Differences in antibody kinetics are also observed when comparing different vaccines (mRNA- vs. vector-based), suggesting that the humoral response depends on the initial pathway through which the vaccine triggers antibody production. This has been evident in the early studies of COVID-19 vaccines [67,68].

The effect of booster doses on antibody kinetics can be explained by the restimulation of the aforementioned mechanisms, which generates an effective immune response against SARS-CoV-2. It can be extrapolated that the enhanced humoral response to COVID-19 vaccines in individuals with prior COVID-19 infection is an analogous phenomenon. In this case, the initial vaccination acts as a booster to the pre-existing humoral response from the infection.

Age is perhaps the most prominent variable predicting the response to COVID-19 vaccines. Most of the studies we analysed indicated that the older population exhibits a weaker immune response to both primary and booster vaccinations. Several studies offer explanations for this relationship. Labrie et al. and Miller and Allman demonstrated in animal models that B-cell production in bone marrow slows with age [69,70]. Frasca et al. found that older individuals have fewer B-cells in systemic circulation, while Shankwitz et al. showed more limited germinal centre responses in older animal models [71,72]. Furthermore, Mogilenko et al. observed age-related alterations in cellular signalling pathways, and Sohrabi et al. noted a reduced number of innate immune cells in older populations [73,74]. These factors likely account for the age-dependent decline in antibody levels over time.

While the data regarding the relationship between sex and antibody levels are inconsistent, some studies suggest there may be differences in immune responses between males and females. Evidence shows that sex steroids (progesterone and testosterone) can alter immune cell function by binding to their receptors [75]. Steroid hormones can also influence antibody production, as demonstrated by Robinson and Klein [76]. Some researchers argue that oestradiol may stimulate immunoglobulin synthesis in B-cells, while higher testosterone levels are associated with faster lipid metabolism, which has been linked to reduced humoral responses in men [77,78]. The relationship between humoral immunity induced by COVID-19 vaccination and sex hormones is thoroughly discussed in the study by Anticoli S. et al. [79]. They argue that sex hormones indicated a significant association between high plasma levels of testosterone and high anti-S/RBD plasma concentrations in male healthcare workers suggestive of a positive immunomodulatory effect of testosterone. The study findings provide a basis for further research on the influence of sex hormones on humoral immunity against SARS-CoV-2.

Moreover, it should be noted that the humoral response generated in mucosal tissues after vaccination still needs to be studied. A study by Zurac et al. described the kinetics of IgG and IgA antibody responses among healthcare workers [80]. While analysing pre-vaccination levels, the authors noted that IgG and IgA levels were higher in previously infected individuals compared to non-infected ones (mean pre-vaccination IgG indexes: non-infected: 0.42, previously infected: 3.02; mean pre-vaccination IgA indexes: non-infected: 0.44, previously infected: 2.29). After the first dose, the mean IgG indexes increased to 4.03 in non-infected and 6.86 in previously infected individuals. Mean IgA indexes increased to 3.05 in non-infected and 7.31 in previously infected individuals. After the second dose, mean IgG indexes rose further to 8.13 in non-infected and 9.56 in previously infected individuals. Mean IgA indexes rose further to 8.41 in non-infected and 10.95 in previously infected individuals. The results demonstrate that IgG levels increased significantly post-first dose (up to 12-fold in non-infected males and 11-fold in females). IgA levels followed a similar trend but had lower baseline levels than IgG. Both IgG and IgA showed plateau-like behaviour after the second dose. This suggests that the IgA response to vaccination likely follows similar trends to IgG, which may influence the effectiveness of acquired immunity against SARS-CoV-2.

## 5. Perspectives for Future Research

It should be emphasised that the healthcare worker population was chosen as the target population for this literature narrative review not only because data on antibody kinetics after vaccination with COVID-19 vaccines in this group were available at the earliest possible time, due to the high priority given to vaccination, but also because the working-age population in most cases is not affected by any of the comorbidities that are a major contributing factor to the vaccine’s immunogenicity.

Not only that, but there is some evidence that healthcare workers have a much better control of their chronic diseases than the general population, as demonstrated by Ko et al. [81], which could lead to a better immune response to COVID-19 vaccines in this particular population compared to non-healthcare workers; therefore, a comparison of the immunogenicity of COVID-19 vaccines between chronically ill healthcare workers and the general population with chronic comorbidities would bring new insights.

It is also now known that vaccines against COVID-19 significantly reduce the risk of developing long COVID-19 syndrome, as noted by Catala et al. [82]. We theorise that with better chronic disease control in healthcare workers and a consequently more effective immune response to COVID-19 vaccines, the risk of long-lasting COVID-19 in the vaccinated chronically ill healthcare worker population should be substantially lower compared to the chronically ill general population and that the syndrome should result in milder symptoms if it is developed. This issue should be considered in future studies.

As mentioned in the Introduction, since the beginning of the pandemic, it has become apparent that healthcare workers are at increased risk of contracting COVID-19 [2,3]. As there is still no consensus on the effective antibody titre value that fully protects against COVID-19 disease, we believe that a reasonable prospect for future studies would be to determine the differences in the levels of protective antibodies against COVID-19 between healthcare workers and the general population, as healthcare workers contract COVID-19 more often and may require higher antibody titres to reduce the risk of developing the disease. If sufficient evidence emerged that a higher antibody titre significantly reduces the risk of contracting COVID-19, it would provide a basis to consider new immunoprophylaxis policies for healthcare workers, such as increasing the frequency of revaccination or administering vaccines targeted at the predominant variant.

It should be noted that although some studies have found an advantage of heterogeneous revaccination over homogeneous revaccination, further follow-up is needed to confirm this fact. In particular, as SARS-CoV-2 continues to mutate, this may lead to a reduction in the effectiveness of even variant-specific vaccines. Analysis of the incidence of COVID-19 among healthcare workers and monitoring of antibody dynamics after vaccination with variant-specific vaccines would provide a better understanding of the efficacy and protective effect of currently available vaccines.

## 6. Limitations of the Review

This literature review provides an overview of the overall trends in antibody dynamics after vaccination with COVID-19 vaccines. Although the review contextualises the changes in antibody dynamics based on a number of different studies looking at different classes of antibodies measured in different units of measurement, its main limitation is that it did not perform a systematic standardisation of the antibody units of measurement, which did not allow for a statistical analysis of antibody dynamics. In the authors’ opinion, this aspect would fall under the frame of meta-analysis. The lack of a static comparison of the studies suggests that this study lacks an investigation of the possible causes of heterogeneity in the study results, sensitivity analyses to assess the robustness of the synthesised results, assessments of risk of bias due to missing results, and assessments of certainty (or confidence) in the body of evidence for each outcome assessed.

Note that healthcare workers tend to be a relatively healthy population and that, as mentioned above, healthcare workers may have better control over their chronic diseases, so caution should be exercised when applying the results described above to a broader population spectrum.

However, this review has clarified the trends in antibody dynamics detected in the studies and the factors which greatly impact the dynamics themselves, such as age, comorbidities, history of COVID-19 disease, etc.

## 7. Conclusions

Although the COVID-19 pandemic has been declared over by the WHO, immunoprophylaxis against SARS-CoV-2 remains a critical aspect of daily clinical practice, both in healthcare settings and for the general population. Our review demonstrated that antibody levels against SARS-CoV-2 inevitably decline after vaccination, leaving the question of ongoing booster strategies open. The studies reviewed provided evidence of the effectiveness of booster vaccination, despite differences in age, sex, and prior COVID-19 infection. This suggests that repeated vaccination remains a highly effective method for mitigating the continued threat posed by COVID-19.

## Figures and Tables

**Table 1:** PICO model.

Population	Healthcare Workers Vaccinated Against SARS-CoV-2
Intervention	Vaccination
Comparator	-
Outcome	Antibody titres

## Data Availability

The original contributions presented in this study are included in the article/Supplementary Materials. Further inquiries can be directed to the corresponding author.

## Supplementary Materials

The following supporting information can be downloaded at: https://www.mdpi.com/article/10.3390/vaccines12121419/s1, Table S1: The main data of the publications.

## References

[1] WHO Coronavirus (COVID-19) Dashboard Overview. https://data.who.int/dashboards/covid19/cases?n=c.

[2] CDC COVID-19 Response Team (2020). Characteristics of Health Care Personnel with COVID-19—United States, February 12–April 9, 2020. MMWR Morb. Mortal. Wkly. Rep..

[3] Chen D., Hu C., Su F., Song Q., Wang Z. (2020). Exposure to SARS-CoV-2 in a high transmission setting increases the risk of severe COVID-19 compared with exposure to a low transmission setting?. J. Travel. Med..

[4] Abo-Leyah H., Gallant S., Cassidy D., Giam Y.H., Killick J., Marshall B., Hay G., Snowdon C., Hothersall E.J., Pembridge T. (2021). The protective effect of SARS-CoV-2 antibodies in Scottish healthcare workers. ERJ Open Res..

[5] Sabetian G., Moghadami M., Hashemizadeh Fard Haghighi L., Shahriarirad R., Fallahi M.J., Asmarian N., Moeini Y.S. (2021). COVID-19 infection among healthcare workers: A cross-sectional study in southwest Iran. Virol. J..

[6] Shields A., Faustini S.E., Perez-Toledo M., Jossi S., Aldera E., Allen J.D., Al-Taei S., Backhouse C., Bosworth A., Dunbar L.A. (2020). SARS-CoV-2 seroprevalence and asymptomatic viral carriage in healthcare workers: A cross-sectional study. Thorax..

[7] Al Maskari Z., Al Blushi A., Khamis F., Al Tai A., Al Salmi I., Al Harthi H., Al Saadi M., Al Mughairy A., Gutierrez R., Al Blushi Z. (2021). Characteristics of healthcare workers infected with COVID-19: A cross-sectional observational study. Int. J. Infect. Dis..

[8] Gómez-Ochoa S.A., Franco O.H., Rojas L.Z., Raguindin P.F., Roa-Díaz Z.M., Wyssmann B.M., Romero Guevara S.L., Echeverria L.E., Glisic M., Muka T. (2021). COVID-19 in Health-Care Workers: A Living Systematic Review and Meta-Analysis of Prevalence, Risk Factors, Clinical Characteristics, and Outcomes. Am. J. Epidemiol..

[9] Coronavirus Disease (COVID-19): Vaccine Access and Allocation. https://www.who.int/news-room/questions-and-answers/item/coronavirus-disease-(COVID-19)-vaccine-access-and-allocation.

[10] Hall V.J., Foulkes S., Saei A., Andrews N., Oguti B., Charlett A., Wellington E., Stowe J., Gillson N., Atti A. (2021). COVID-19 vaccine coverage in health-care workers in England and effectiveness of BNT162b2 mRNA vaccine against infection (SIREN): A prospective, multicentre, cohort study. Lancet.

[11] Association Between Vaccination with BNT162b2 and Incidence of Symptomatic and Asymptomatic SARS-CoV-2 Infections Among Health Care Workers. https://pubmed.ncbi.nlm.nih.gov/33956048/.

[12] Bouton T.C., Lodi S., Turcinovic J., Schaeffer B., Weber S.E., Quinn E., Korn C., Steiner J., Schechter-Perkins E.M., Duffy E. (2021). Coronavirus Disease 2019 Vaccine Impact on Rates of Severe Acute Respiratory Syndrome Coronavirus 2 Cases and Postvaccination Strain Sequences Among Health Care Workers at an Urban Academic Medical Center: A Prospective Cohort Study. Open Forum Infect. Dis..

[13] Bergwerk M., Gonen T., Lustig Y., Amit S., Lipsitch M., Cohen C., Mandelboim M., Levin E.G., Rubin C., Indenbaum V. (2021). COVID-19 Breakthrough Infections in Vaccinated Health Care Workers. N. Engl. J. Med..

[14] Patel R., Kaki M., Potluri V.S., Kahar P., Khanna D. (2022). A comprehensive review of SARS-CoV-2 vaccines: Pfizer, Moderna & Johnson & Johnson. Hum. Vaccines Immunother..

[15] Cegolon L., Magnano G., Negro C., Larese Filon F., ORCHESTRA Working Group (2023). SARS-CoV-2 Reinfections in Health-Care Workers, 1 March 2020–31 January 2023. Viruses.

[16] Janke C., Rubio-Acero R., Weigert M., Reinkemeyer C., Khazaei Y., Kleinlein L., Le Gleut R., Radon K., Hannes M., Picasso F. (2024). Understanding the Omicron Variant Impact in Healthcare Workers: Insights from the Prospective COVID-19 Post-Immunization Serological Cohort in Munich (KoCo-Impf) on Risk Factors for Breakthrough and Reinfections. Viruses.

[17] Moher D., Liberati A., Tetzlaff J., Altman D.G. (2010). Preferred reporting items for systematic reviews and meta-analyses: The PRISMA statement. Int. J. Surg..

[18] Anastassopoulou C., Antoni D., Manoussopoulos Y., Stefanou P., Argyropoulou S., Vrioni G., Tsakris A. (2022). Age and sex associations of SARS-CoV-2 antibody responses post BNT162b2 vaccination in healthcare workers: A mixed effects model across two vaccination periods. PLoS ONE.

[19] Oliveira-Silva J., Reis T., Lopes C., Batista-Silva R., Ribeiro R., Marques G., Pacheco V., Rodrigues T., Afonso A., Pinheiro V. (2022). Humoral response to the SARS-CoV-2 BNT162b2 mRNA vaccine: Real-world data from a large cohort of healthcare workers. Vaccine.

[20] Salvagno G.L., Henry B.M., Pighi L., De Nitto S., Gianfilippi G.L., Lippi G. (2021). Three-month analysis of total humoral response to Pfizer BNT162b2 mRNA COVID-19 vaccination in healthcare workers. J. Infect..

[21] Van Elslande J., Weemaes M., Godderis L., Van Pottelbergh G., Bossuyt X., Vermeersch P. (2022). IgG anti-spike antibody levels in healthcare workers with and without prior COVID-19 up to 3 months after BNT162b2 vaccination. Diagn. Microbiol. Infect. Dis..

[22] Visci G., Zunarelli C., Mansour I., Porru S., De Palma G., Duval X., Monaco M.G.L., Spiteri G., Carta A., Lippi G. (2022). Serological response after SARS-CoV2 vaccination in healthcare workers: A multicenter study. Med. Lav..

[23] IgG Antibodies Against SARS-CoV-2 Decay But Persist 4 Months After Vaccination in a Cohort of Healthcare Workers. https://pubmed.ncbi.nlm.nih.gov/34755649/.

[24] Cangemi R., Di Franco M., Angeloni A., Zicari A., Cardinale V., Visentini M., Antonelli G., Napoli A., Anastasi E., Romiti G.F. (2022). Serological Response and Relationship with Gender-Sensitive Variables among Healthcare Workers after SARS-CoV-2 Vaccination. J. Pers. Med..

[25] Bayart J.L., Douxfils J., Gillot C., David C., Mullier F., Elsen M., Eucher C., Van Eeckhoudt S., Roy T., Gerin V. (2021). Waning of IgG, Total and Neutralizing Antibodies 6 Months Post-Vaccination with BNT162b2 in Healthcare Workers. Vaccines.

[26] Collatuzzo G., Visci G., Violante F.S., Porru S., Spiteri G., Monaco M.G.L., Fillon F.L., Negro C., Janke C., Castelletti N. (2022). Determinants of anti-S immune response at 6 months after COVID-19 vaccination in a multicentric European cohort of healthcare workers—ORCHESTRA project. Front. Immunol..

[27] Đaković Rode O., Bodulić K., Zember S., Cetinić Balent N., Novokmet A., Čulo M., Rašić Ž., Mikulić R., Markotić A. (2022). Decline of Anti-SARS-CoV-2 IgG Antibody Levels 6 Months after Complete BNT162b2 Vaccination in Healthcare Workers to Levels Observed Following the First Vaccine Dose. Vaccines.

[28] Krintus M., Piasecki M., Lackowski P., Buszko K., Kubica A., Kosobucka-Ozdoba A., Michalski P., Pietrzykowski L., Stolarek W., Wojcik A. (2022). Determinants of the Level of Anti-SARS-CoV-2 IgG ANTibodiEs after Vaccination (DANTE-SIRIO 7) Study in a Large Cohort of Healthcare Workers. Vaccines.

[29] Fernández-Rivas G., Barallat J., Quirant-Sánchez B., González V., Doladé M., Martinez-Caceres E., Piña M., Matllo J., Blanco I., Cardona P.J. (2022). Follow up of the Humoral Response in Healthcare Workers after the Administration of Two Dose of the Anti SARS-CoV-2 Vaccines-Effectiveness in Delta Variant Breakthrough Infections. Viruses.

[30] Infantino M., Manfredi M., Stacchini L., Cosma C., Grossi V., Lari B., Russo E., Amedei A., Benucci M., Veneziani F. (2022). The role of neutralizing antibodies by sVNT after two doses of BNT162b2 mRNA vaccine in a cohort of Italian healthcare workers. Clin. Chem. Lab. Med..

[31] Oliveira-Silva J., Reis T., Lopes C., Batista-Silva R., Ribeiro R., Marques G., Pacheco V., Rodrigues T., Afonso A., Pinheiro V. (2022). Long-term serological SARS-CoV-2 IgG kinetics following mRNA COVID-19 vaccine: Real-world data from a large cohort of healthcare workers. Int. J. Infect. Dis..

[32] Ramos A., Cardoso M.J., Ribeiro L., Guimarães J.T. (2022). Assessing SARS-CoV-2 Neutralizing Antibodies after BNT162b2 Vaccination and Their Correlation with SARS-CoV-2 IgG Anti-S1, Anti-RBD and Anti-S2 Serological Titers. Diagnostics.

[33] Mueller T. (2022). Time course of antibody concentrations against the spike protein of SARS-CoV-2 among healthy hospital workers up to 200 days after their first COVID-19 vaccination. J. Clin. Lab. Anal..

[34] Gil-Manso S., Alonso R., Catalán P., Sánchez-Arcilla I., Marzola M., Correa-Rocha R., Pion M., Muñoz P. (2022). IgG anti-RBD levels during 8-month follow-up post-vaccination with BNT162b2 and mRNA-1273 vaccines in healthcare workers: A one-center study. Front. Cell. Infect. Microbiol..

[35] Golec M., Fronczek M., Zembala-John J., Chrapiec M., Konka A., Wystyrk K., Botor H., Brzoza Z., Kasperczyk S., Bułdak R.J. (2022). Early and Longitudinal Humoral Response to the SARS-CoV-2 mRNA BNT162b2 Vaccine in Healthcare Workers: Significance of BMI, Adipose Tissue and Muscle Mass on Long-Lasting Post-Vaccinal Immunity. Viruses.

[36] Serrano L., Algarate S., Herrero-Cortina B., Bueno J., González-Barriga M.T., Ducons M., Montero-Marco J., Acha B., Taboada A., Sanz-Burillo P. (2022). Assessment of humoral immune response to two mRNA SARS-CoV-2 vaccines (Moderna and Pfizer) in healthcare workers fully vaccinated with and without a history of previous infection. J. Appl. Microbiol..

[37] Wolszczak-Biedrzycka B., Bieńkowska A., Dorf J. (2021). Assessment of Post-Vaccination Antibody Response Eight Months after the Administration of BNT1622b2 Vaccine to Healthcare Workers with Particular Emphasis on the Impact of Previous COVID-19 Infection. Vaccines.

[38] Inchingolo A.D., Malcangi G., Ceci S., Patano A., Corriero A., Azzollini D., Marinelli G., Coloccia G., Piras F., Barile G. (2022). Antispike Immunoglobulin-G (IgG) Titer Response of SARS-CoV-2 mRNA-Vaccine (BNT162b2): A Monitoring Study on Healthcare Workers. Biomedicines.

[39] Collatuzzo G., De Palma G., Violante F.S., Porru S., Filon F.L., Fabianova E., Violan C., Vimercati L., Leustean M., Rodriguez-Suarez M.M. (2023). Corrigendum: Temporal trends of COVID-19 antibodies in vaccinated healthcare workers undergoing repeated serological sampling: An individual-level analysis within 13 months in the ORCHESTRA cohort. Front. Immunol..

[40] Lorent D., Nowak R., Figlerowicz M., Handschuh L., Zmora P. (2024). Anti-SARS-CoV-2 Antibodies Level and COVID-19 Vaccine Boosters among Healthcare Workers with the Highest SARS-CoV-2 Infection Risk-Follow Up Study. Vaccines.

[41] Stocchi M., Melodia P., Lucini A., De Lorenzo R., Pozzi C., Rovere-Querini P., Odone A., Renzi C., Signorelli C. (2024). COVID-19 Immunity in the Cohort of IRCCS San Raffaele Hospital Employees after BNT162b2 Vaccination: A Retrospective Observational Study. Ann. Ig. Med. Prev. E Comunita..

[42] Chivu-Economescu M., Vremera T., Ruta S.M., Grancea C., Leustean M., Chiriac D., David A., Matei L., Diaconu C.C., Gatea A. (2022). Assessment of the Humoral Immune Response Following COVID-19 Vaccination in Healthcare Workers: A One Year Longitudinal Study. Biomedicines.

[43] Long Follow-Up of BNT162b2 mRNA Vaccine in Healthcare Workers (2020–2022): A Retrospective Longitudinal SARS-CoV-2 Serological Surveillance. https://pubmed.ncbi.nlm.nih.gov/37724517/.

[44] Guibert N., Trepat K., Pozzetto B., Josset L., Fassier J.B., Allatif O., Saker K., Brengel-Pesce K., Walzer T., Vanhems P. (2023). A third vaccine dose equalises the levels of effectiveness and immunogenicity of heterologous or homologous COVID-19 vaccine regimens, Lyon, France, December 2021 to March 2022. Eurosurveillance.

[45] Leomanni L., Collatuzzo G., Sansone E., Sala E., De Palma G., Porru S., Spiteri G., Monaco M.G.L., Basso D., Pavanello S. (2023). Determinants of Anti-S Immune Response at 12 Months after SARS-CoV-2 Vaccination in a Multicentric European Cohort of Healthcare Workers-ORCHESTRA Project. Vaccines.

[46] Padoan A., Cosma C., Della Rocca F., Barbaro F., Santarossa C., Dall’Olmo L., Galla L., Cattelan A., Cianci V., Basso D. (2022). A cohort analysis of SARS-CoV-2 anti-spike protein receptor binding domain (RBD) IgG levels and neutralizing antibodies in fully vaccinated healthcare workers. Clin. Chem. Lab. Med..

[47] Pavlidis G., Giannoulis V., Pirounaki M., Lampropoulos I.C., Siafi E., Nitsa A., Pavlou E., Xanthaki A., Perlepe G., Fortis S.P. (2023). Evaluation of Antibody Kinetics Following COVID-19 Vaccination in Greek SARS-CoV-2 Infected and Naïve Healthcare Workers. J. Pers. Med..

[48] Skorupa M., Szczepanek J., Goroncy A., Jarkiewicz-Tretyn J., Ptaszyńska B., Rajewski P., Koper W., Pałgan K., Tretyn K. (2022). The Dynamics of Changes in the Concentration of IgG against the S1 Subunit in Polish Healthcare Workers in the Period from 1 to 12 Months after Injection, Including Four COVID-19 Vaccines. Vaccines.

[49] Vietri M.T., D’Elia G., Caliendo G., Passariello L., Albanese L., Molinari A.M., Angelillo I.F. (2022). Antibody levels after BNT162b2 vaccine booster and SARS-CoV-2 Omicron infection. Vaccine.

[50] Gerhards C., Thiaucourt M., Hetjens M., Haselmann V., Neumaier M., Kittel M. (2023). Heterologous Vector-mRNA Based SARS-CoV-2 Vaccination Strategy Appears Superior to a Homologous Vector-Based Vaccination Scheme in German Healthcare Workers Regarding Humoral SARS-CoV-2 Response Indicating a High Boosting Effect by mRNA Vaccines. Vaccines.

[51] Grassi T., Lobreglio G., Panico A., Rosato C., Zizza A., Lazzari R., Chicone M., Indino F., Bagordo F. (2022). Kinetics of Humoral Immunity against SARS-CoV-2 in Healthcare Workers after the Third Dose of BNT162b2 mRNA Vaccine. Vaccines.

[52] Skrzat-Klapaczyńska A., Kowalska J.D., Paciorek M., Puła J., Bieńkowski C., Krogulec D., Stengiel J., Pawełczyk A., Perlejewski K., Osuch S. (2022). Higher Immunological Response after BNT162b2 Vaccination among COVID-19 Convalescents-The Data from the Study among Healthcare Workers in an Infectious Diseases Center. Vaccines.

[53] Consonni D., Lombardi A., Mangioni D., Bono P., Oggioni M., Uceda Renteria S., Valzano A., Bordini L., Nava C.D., Tiwana N. (2022). Immunogenicity and effectiveness of BNT162b2 COVID-19 vaccine in a cohort of healthcare workers in Milan (Lombardy Region, Northern Italy). Epidemiol. Prev..

[54] Augustinussen M.H., Tylden G.D., Rinaldo C.H. (2023). Dynamics of SARS-CoV-2 Spike-IgG throughout Three COVID-19 Vaccination Regimens: A 21-Month Longitudinal Study of 82 Norwegian Healthcare Workers. Viruses.

[55] Łysek-Gładysińska M., Starz M., Borowiec-Sęk A., Sufin I., Wieczorek A., Chrapek M., Zarębska-Michaluk D., Sufin P., Głuszek S., Adamus-Białek W. (2023). The Levels of Anti-SARS-CoV-2 Spike Protein IgG Antibodies Before and After the Third Dose of Vaccination Against COVID-19. J. Inflamm. Res..

[56] Serra N., Andriolo M., Butera I., Mazzola G., Sergi C.M., Fasciana T.M.A., Giammanco A., Gagliano M.C., Cascio A., Di Carlo P. (2023). A Serological Analysis of the Humoral Immune Responses of Anti-RBD IgG, Anti-S1 IgG, and Anti-S2 IgG Levels Correlated to Anti-N IgG Positivity and Negativity in Sicilian Healthcare Workers (HCWs) with Third Doses of the mRNA-Based SARS-CoV-2 Vaccine: A Retrospective Cohort Study. Vaccines.

[57] Isgrò M.A., Trillò G., Russo L., Tornesello A.L., Buonaguro L., Tornesello M.L., Miscio L., Normanno N., Bianchi A.A.M., Buonaguro F.M. (2022). Bimodal antibody-titer decline following BNT162b2 mRNA anti-SARS-CoV-2 vaccination in healthcare workers of the INT—IRCCS “Fondazione Pascale” Cancer Center (Naples, Italy). Infect. Agent. Cancer.

[58] Sarrigeorgiou I., Moschandreou D., Dimitriadis A., Tsinti G., Sotiropoulou E., Ntoukaki E., Eliadis P., Backovic M., Labropoulou S., Escriou N. (2022). Combined monitoring of IgG and IgA anti-Spike and anti-Receptor binding domain long term responses following BNT162b2 mRNA vaccination in Greek healthcare workers. PLoS ONE.

[59] Zurac S., Vladan C., Dinca O., Constantin C., Neagu M. (2022). Immunogenicity evaluation after BNT162b2 booster vaccination in healthcare workers. Sci. Rep..

[60] Szczepanek J., Skorupa M., Jarkiewicz-Tretyn J., Tretyn A. (2023). COVID-19 vaccination in healthcare workers: Long-term benefits and protection. Cent-Eur. J. Immunol..

[61] Scheiermann N., Kuwert E.K. (1983). Uptake and elimination of hepatitis B immunoglobulins after intramuscular application in man. Dev. Biol. Stand..

[62] Hopkins R.J., Kramer W.G., Blackwelder W.C., Ashtekar M., Hague L., Winker-La Roche S.D., Berezuk G., Smith D., Leese P.T. (2004). Safety and pharmacokinetic evaluation of intravenous vaccinia immune globulin in healthy volunteers. Clin. Infect. Dis..

[63] Adner N., Leibl H., Enzersberger O., Kirgios M., Wahlberg T. (2001). Pharmacokinetics of human tick-borne encephalitis virus antibody levels after injection with human tick-borne encephalitis immunoglobulin, solvent/detergent treated, FSME-BULIN S/D in healthy volunteers. Scand. J. Infect. Dis..

[64] Ghetie V., Ward E.S. (2002). Transcytosis and catabolism of antibody. Immunol. Res..

[65] Amanna I.J., Slifka M.K. (2010). Mechanisms that determine plasma cell lifespan and the duration of humoral immunity. Immunol. Rev..

[66] Farber D.L., Netea M.G., Radbruch A., Rajewsky K., Zinkernagel R.M. (2016). Immunological memory: Lessons from the past and a look to the future. Nat. Rev. Immunol..

[67] Sahin U., Muik A., Derhovanessian E., Vogler I., Kranz L.M., Vormehr M., Baum A., Pascal K., Quandt J., Maurus D. (2020). COVID-19 vaccine BNT162b1 elicits human antibody and TH1 T cell responses. Nature.

[68] Voysey M., Clemens S.A.C., Madhi S.A., Weckx L.Y., Folegatti P.M., Aley P.K., Angus B., Baillie V.L., Barnabas S.L., Bhorat Q.E. (2021). Safety and efficacy of the ChAdOx1 nCoV-19 vaccine (AZD1222) against SARS-CoV-2: An interim analysis of four randomised controlled trials in Brazil, South Africa, and the UK. Lancet.

[69] Labrie J.E., Sah A.P., Allman D.M., Cancro M.P., Gerstein R.M. (2004). Bone Marrow Microenvironmental Changes Underlie Reduced RAG-mediated Recombination and B Cell Generation in Aged Mice. J. Exp. Med..

[70] Miller J.P., Allman D. (2003). The decline in B lymphopoiesis in aged mice reflects loss of very early B-lineage precursors. J. Immunol..

[71] Frasca D., Diaz A., Romero M., Landin A.M., Blomberg B.B. (2011). Age effects on B cells and humoral immunity in humans. Ageing Res. Rev..

[72] Shankwitz K., Pallikkuth S., Sirupangi T., Kirk Kvistad D., Russel K.B., Pahwa R., Gama L., Koup R.A., Pan L., Villinger F. (2020). Compromised steady-state germinal center activity with age in nonhuman primates. Aging Cell.

[73] Mogilenko D.A., Shchukina I., Artyomov M.N. (2022). Immune ageing at single-cell resolution. Nat. Rev. Immunol..

[74] Sohrabi Y., Reinecke H., Joosten L.A.B., Netea M.G. (2021). Deadly COVID-19 among the elderly: Innate immune memory helping those most in need. Med.

[75] Kovats S., Carreras E., Agrawal H., Klein S.L., Roberts C. (2010). Sex Steroid Receptors in Immune Cells. Sex Hormones and Immunity to Infection.

[76] Robinson D.P., Klein S.L. (2012). Pregnancy and pregnancy-associated hormones alter immune responses and disease pathogenesis. Horm. Behav..

[77] Lü F.X., Abel K., Ma Z., Rourke T., Lu D., Torten J., McChesney M., Miller C.J. (2002). The strength of B cell immunity in female rhesus macaques is controlled by CD8+ T cells under the influence of ovarian steroid hormones. Clin. Exp. Immunol..

[78] Furman D., Hejblum B.P., Simon N., Jojic V., Dekker C.L., Thiébaut R., Tibshirani R.J., Davis M.M. (2014). Systems analysis of sex differences reveals an immunosuppressive role for testosterone in the response to influenza vaccination. Proc. Natl. Acad. Sci. USA.

[79] Anticoli S., Dorrucci M., Iessi E., Chiarotti F., Di Prinzio R.R., Vinci M.R., Zaffina S., Puro V., Colavita F., Mizzoni K. (2023). Association between sex hormones and anti-S/RBD antibody responses to COVID-19 vaccines in healthcare workers. Hum. Vaccines Immunother..

[80] Zurac S., Nichita L., Mateescu B., Mogodici C., Bastian A., Popp C., Cioplea M., Socoliu C., Constantin C., Neagu M. (2021). COVID-19 vaccination and IgG and IgA antibody dynamics in healthcare workers. Mol. Med. Rep..

[81] Ko D.T., Chu A., Austin P.C., Johnston S., Nallamothu B.K., Roifman I., Tusevljak N., Udell J.A., Frank E. (2019). Comparison of Cardiovascular Risk Factors and Outcomes Among Practicing Physicians vs the General Population in Ontario, Canada. JAMA Netw. Open.

[82] Català M., Mercadé-Besora N., Kolde R., Trinh N.T.H., Roel E., Burn E., Rathod-Mistry T., Kostka K., Man W.Y., Delmestri A. (2024). The effectiveness of COVID-19 vaccines to prevent long COVID symptoms: Staggered cohort study of data from the UK, Spain, and Estonia. Lancet Respir. Med..

